# Estrogens Attenuate Oxidative Stress and the Differentiation and Apoptosis of Osteoblasts by DNA-Binding-Independent Actions of the ERα

**DOI:** 10.1359/jbmr.091017

**Published:** 2009-10-12

**Authors:** Maria Almeida, Marta Martin-Millan, Elena Ambrogini, Robert Bradsher, Li Han, Xiao-Dong Chen, Paula K Roberson, Robert S Weinstein, Charles A O'Brien, Robert L Jilka, Stavros C Manolagas

**Affiliations:** Division of Endocrinology and Metabolism, Center for Osteoporosis and Metabolic Bone Diseases, University of Arkansas for Medical Sciences and the Central Arkansas Veterans Health Care SystemLittle Rock, AR, USA

**Keywords:** reactive oxygen species, p66^shc^, ERKs, BMP-2, estrogen receptor

## Abstract

Estrogens diminish oxidative stress in bone and bone marrow, attenuate the generation of osteoblasts, and decrease the prevalence of mature osteoblast apoptosis. We have searched for the molecular mechanism of these effects using as tools a mouse model bearing an estrogen receptor α (*ERα*) knock-in mutation that prevents binding to DNA (*ERα*^*NERKI*/−^) and several osteoblast progenitor cell models expressing the wild-type *ERα* or the *ERα*^*NERKI*/−^. We report that the ability of estrogens to diminish the generation of reactive oxygen species, stimulate the activity of glutathione reductase, and decrease the phosphorylation of p66^shc^, as well as osteoblastogenesis and osteoblast number and apoptosis, were fully preserved in *ERα*^*NERKI*/−^ mice, indicating that the DNA-binding function of the ERα is dispensable for all these effects. Consistent with the attenuation of osteoblastogenesis in this animal model, 17β-estradiol attenuated bone morphogenetic protein 2 (BMP-2)–induced gene transcription and osteoblast commitment and differentiation in murine and human osteoblastic cell lines. Moreover, 17β-estradiol attenuated BMP-2-induced differentiation of primary cultures of calvaria- or bone marrow–derived osteoblastic cells from *ERα*^*NERKI*/−^ mice as effectively as in cells from wild-type littermates. The inhibitory effect of the hormone on BMP-2 signaling resulted from an ERα-mediated activation of ERKs and the phosphorylation of Smad1 at the linker region of the protein, which leads to proteasomal degradation. These results illustrate that the effects of estrogens on oxidative stress and the birth and death of osteoblasts do not require the binding of ERα to DNA response elements, but instead they result from the activation of cytoplasmic kinases. © 2010 American Society for Bone and Mineral Research

## Introduction

Work from our group during the last 20 years has elucidated that estrogens protect the adult skeleton against bone loss by slowing the rate of bone remodeling and by maintaining a focal balance between bone formation and resorption.(1–3) Slowing of bone remodeling results from the attenuating effects of sex steroids on the birth rate of osteoclast and osteoblast progenitors.(4,5) Maintenance of a focal balance between formation and resorption results from opposite effects on the lifespan of osteoclasts and osteoblasts/osteocytes: a proapoptotic effect on osteoclasts and an antiapoptotic effect on osteoblasts and osteocytes.(6–8) The effects of estrogens on osteoclast and osteoblast apoptosis are exerted by a mechanism that is distinct from that requiring direct interaction of their receptors with DNA (hormone-response element) or protein-protein interaction between the receptor and other transcription factors. Instead, the effect of estrogens on the apoptosis of either cell type is the result of an extranuclear action of the classical receptors that cause activation of cytoplasmic kinases, including extracellular signal-regulated kinases (ERKs) and kinase-dependent changes in the activity of transcription factors.(6,8,9) The mechanistic basis for the divergence of estrogens' effect on the survival of the two cell types downstream from ERKs is evidently dependent on the kinetics of ERK phosphorylation and the length of time that phospho-ERKs are retained in the nucleus, perhaps by determining the activation of a distinct set of transcription factors.(9)

We have demonstrated previously that the number of osteoblast progenitors, as measured by colony-forming units–osteoblast (CFU-OB), increase after loss of estrogens in mice(10) and that this change is partially preserved in mice treated with bisphosphonates, which significantly decrease osteoclast number, strongly suggesting that bone resorption (and the release of growth factors from the bone matrix) is not required for the increase in osteoblast precursors. Therefore, estrogens must suppress osteoblastogenesis by direct actions on early osteoblast precursors. Further, we have shown that most CFU-OBs are early transit-amplifying progenitors (i.e., dividing cells with limited self-renewal capacity) and that their replication is indeed attenuated by estrogens.(5)

We and others also have shown previously that estrogens attenuate the transcription of bone morphogenetic protein 2 (BMP-2) target genes.(11–13) BMPs are members of the transforming growth factor β (TGF-β) superfamily and play an essential role in skeletal development and repair.(14,15) Specifically, BMPs promote embryonic and postnatal osteogenesis by inducing the commitment of mesenchymal cells to the osteoblastic lineage and promoting osteoblast differentiation.(16,17) Binding of BMPs to their receptor serine/threonine kinases results in the phosphorylation of Smads 1, 5, and 8(18) at the carboxy terminus and translocation into the nucleus after heterodimerization with Smad4. In the nucleus, the complex either binds to DNA sequences directly or can interact with several transcription factors to control the activity of hundreds of downstream target genes.(19,20) The Smad proteins consist of two globular domains (MH1 and MH2 domains) connected by a linker region. In Smad 1, 5, and 8, the latter contains four MAPK phosphorylation sites and two putative GSK-3β sites.(19,21) Importantly, MAPK phosphorylation of the linker region inhibits Smad function and therefore BMP-induced transcription both in vitro and in vivo.(22–24)

More recently, we and others have obtained evidence that the protective effects of estrogens on bone result from their ability to attenuate oxidative stress and that loss of estrogens accelerates the effects of aging. Specifically, we have shown that C57BL/6 mice lose bone strength and mass progressively between the ages of 4 and 31 months(25) and that these changes are temporally associated with decreased osteoblast and osteoclast numbers and decreased bone-formation rate as well as increased osteoblast and osteocyte apoptosis. These changes are also temporally linked with increased reactive oxygen species (ROS) levels and decreased glutathione reductase (GSR) activity in the bone marrow, as well as a corresponding increase in the phosphorylation of p66^shc^—an adapter protein that serves as a key component of a signaling cascade that is activated by ROS and influences apoptosis and lifespan in invertebrates and mammals.(26) Indeed, proapoptotic signals, including ROS, release p66^shc^ from an inhibitory complex, and active p66^shc^ serves as a redox enzyme that catalyzes reduction of O_2_ to H_2_O_2_ through electron transfer from cytochrome c. H_2_O_2_, in turn, causes opening of the mitochondrial permeability transition pore, swelling, and apoptosis. An increase in ROS production and p66^shc^ phosphorylation, as well as decreased GSR activity, was reproduced acutely in our previous work by gonadectomy in either female or male C57BL/6 mice and prevented by the antioxidant *N*-acetyl-cysteine (NAC).(25) In agreement with our in vivo findings, results of in vitro experiments demonstrated that the effects of estrogens on osteoclastogenesis and osteoclast and osteoblast apoptosis result from cell autonomous antioxidant actions of the hormone on the respective cell types, are dependent on the estrogen receptor (ER), and are mediated via ERKs. Moreover, we have shown that estrogens attenuate the phosphorylation of p66^shc^ in osteoblastic cells and that this effect is also mediated via ERKs. Based on these findings, we have proposed that loss of estrogens accelerates the effects of aging on bone by decreasing the defense against oxidative stress.

In this study we have investigated the molecular actions of ERα on osteoblasts using as a tool a mouse model bearing an *ERα* knock-in mutation that prevents binding to DNA (*ERα*^*NERKI*/−^).(27) We previously determined that *ERα*^*NERKI*/−^ mice have an atrophic uterus despite normal estrogen levels and that estrogen replacement does not restore it in ovariectomized (OVX) *ERα*^*NERKI*/−^ mice, but it does induce the activation of ERKs and the ERK-mediated phosphorylation of the transcription factor Elk-1 in vertebrae.(13) In addition, in this study we have investigated in vitro the signaling cascades downstream from ERα that are responsible for its effects on osteoblast commitment and differentiation. We show that the effects of estrogens on oxidative stress and the birth and death of osteoblasts are fully preserved in *ERα*^*NERKI*/−^ mice. Consistent with the attenuation of osteoblastogenesis in the *ERα*^*NERKI*/−^ mice, 17β-estradiol (E_2_) attenuates BMP-2-induced gene transcription and differentiation of preosteoblastic cell lines as well as primary cultures of calvaria- or bone marrow–derived osteoblastic cells from *ERα*^*NERKI*/−^ mice as effectively as in cells from wild-type littermates. This effect is due to an ERα-mediated activation of ERKs and the phosphorylation of Smad1 at the linker region of the protein, which leads to proteasomal degradation.

## Materials and Methods

### Chemicals, reagents, and plasmids

Etoposide, H_2_O_2_, and E_2_ were purchased from Sigma-Aldrich (St. Louis, MO, USA). BMP-2 and fibroblast growth factor 2 (FGF2) recombinant proteins were purchased from R&D Systems (Minneapolis, MN, USA). PD98059 was purchased from Cell Signaling Technology, Inc. (Danvers, MA, USA). The BMP-responsive luciferase reporter construct (BRE)-Luc was obtained from Peter ten Dijke (Leiden University Medical Center, Leiden, The Netherlands).(28)

### Animal experimentation

Mice heterozygous for an *ERα* knock-in mutant in a 129SvJ background were provided by J Larry Jameson (Northwestern University, Chicago, IL, USA).(27) Mice harboring an inactivating mutation in the *ERα* locus (*ERα*^+/−^) in a C57BL/6 background were provided by Andree Krust and Pierre Chambon (Institute for Genetics and Cellular and Molecular Biology, Strasbourg, France).(29) *ERα*^*NERKI*/+^ mice were crossed with heterozygous *ERα*^+/−^ female mice to produce animals carrying only one *NERKI* allele (*ERα*^*NERKI*/−^). Five-month-old female *ERα*^*NERKI*/−^ mice of the F_1_ generation and their *ERα*^*NERKI*/+^, *ERα*^+/−^, and *ERα*^+/+^ littermates were electronically tagged (Biomedic Data System, Inc., Maywood, NJ, USA), and bone mineral density (BMD) measurements were performed on each mouse. Animals then were sham-operated or ovariectomized (OVX). The following day, OVX animals were subcutaneously injected with vehicle or with replacement doses of E_2_ (30 ng/g/day; *n* = 12 per group), and sham-operated animals were injected with vehicle. After 6 weeks of treatment, animals were sacrificed and the tissues dissected for further analyses. BMD, vertebral dimensions, osteoblast number, and osteoblast apoptosis were obtained as described previously.(7,30,31) *ERα*^−/−^ and corresponding wild-type (WT) littermate control mice were generated by crossing heterozygous *ERα*^+/−^ mice.

### Western blot analysis

The phosphorylation status of p66^shc^ was analyzed by immunoblotting in fifth lumbar vertebra lysates, as described previously,(8) using a mouse monoclonal antibody recognizing Ser36 phosphorylated p66^shc^ (Calbiochem, San Diego, CA, USA). Protein levels of p66^shc^ were analyzed using a rabbit polyclonal antibody recognizing p66^shc^ (BD Biosciences, Palo Alto, CA, USA). The antibody recognizing p-Smad1/5/8 was purchased from Cell Signaling. The antibody recognizing phospho-Smad1 (Ser214) was kindly provided by EM De Robertis (Howard Hughes Medical Institute and University of California, Los Angeles, CA, USA).(32) The antibodies against Smad4, p-ERK1/2, and ERK1/2 were purchased from Santa Cruz Biotechnology (Santa Cruz, CA, USA). Quantification of the intensity of the bands in the autoradiograms was performed using a VersaDoc imaging system (Bio-Rad Laboratories, Hercules, CA, USA).

### Micro-Computed Tomography (µCT)

µCT analysis of the sixth lumbar vertebrae was done after the bones were dissected, cleaned, fixed in 10% Millonig's formalin, transferred to ethanol, loaded into 12.3 mm diameter scanning tubes, and imaged (µCT40, Scanco Medical, Basserdorf, Switzerland). Scans were integrated into 3D voxel images (1024 × 1024 pixel matrices for each individual planar stack), and a Gaussian filter (σ = 0.8, support = 1) was used to reduce signal noise. A threshold of 200 was applied to all analyzed scans. Scans were done at medium resolution (*E* = 55 kVp, *I* = 145 µA, integration time = 200 ms). The entire vertebral body was scanned with a transverse orientation excluding the pedicles and articular processes. Manual analysis excluded the cortical bone and the primary spongiosa from the analysis. All trabecular measurements were made by manually drawing contours every 10 to 20 slices and using voxel counting for bone volume per tissue volume and sphere-filling distance-transformation indices without assumptions about the bone shape as a rod or plate for trabecular microarchitecture. Cortical thickness was measured at the tibial mid-diaphysis.

### Cell culture, transfections, and luciferase activity

Osteoblastic cells derived from mouse calvaria or bone marrow were obtained and cultured as described previously,(33) and during exposure to E_2_, the cultures were maintained in 2% charcoal-stripped serum. Osteoblast differentiation was analyzed using freshly isolated cells cultured in 12 well tissue culture plates at 5 × 10^6^ cells per well in α modified essential medium (α-MEM) containing 10% fetal bovine serum (FBS) for 10 days. Half the medium was replaced every 5 days. FBS then was reduced to 2%, and 10^−8^ M E_2_ was added in the presence or absence of 25 ng/mL BMP-2. Two days later, 10 mM β-glycerophosphate was added to the medium, and the cultures were maintained for an additional 2 weeks. The mineralized matrix was stained with 40 mM alizarin red, pH 4.2. Alizarin red was quantified after extraction with 10 mM sodium phosphate, 10% cetylpyridinium chloride, pH 7, and absorbance determination at 562 nm against a known alizarin red standard. For assay of caspase 3 activity, the medium was changed to serum-free prior to the addition of the different compounds. Colony-forming units–fibroblast (CFU-F) and CFU-OB number were determined as described previously,(5) using guinea pig feeder cells,(34) 15% FBS, and 1 mM ascorbate-2-phosphate. Half the medium was replaced every 5 days. CFU-Fs were enumerated at 10 days of culture after staining for alkaline phosphatase, and CFU-OBs were enumerated at 25 days of culture after von Kossa staining. Colonies containing more than 50 fibroblastic cells were enumerated and plotted as a function of the number of cells seeded. C2C12 and 2T3 cells were maintained in Dulbecco's modified Eagle's medium supplemented with 10% FBS and 1% each of penicillin, streptomycin, and glutamine and 1% sodium pyruvate. U2OS cells stably expressing tetracycline-inducible ERα (U2OS-ERα) were kindly provided by DC Leitman (University of California, San Francisco, CA, USA).(35) U2OS-ERα cells were maintained in phenol red–free McCoy medium supplemented with 10% FBS and 1% each of penicillin, streptomycin, and glutamine. Cells were incubated for 24 hours with or without doxycycline (1 µg/mL) and serum starved for another 16 hours previous to the addition of BMP-2 or E_2_. Mouse embryonic fibroblasts (MEFs) from WT or *Smad1*^*L/L*^ mice, kindly provided by P Soriano (Fred Hutchinson Cancer Research Center, Seattle, WA, USA),(24) were cultured in DMEM supplemented with 10% FBS. Plasmid constructs were introduced into cells by transient transfection using Lipofectamine Plus (Invitrogen, Carlsbad, CA. USA). Cells were plated in 48 well plates and transfected 16 hours later with a total of 0.4 µg of DNA. Luciferase activity assays were performed as described previously.(36)

### Alkaline phosphatase (AP) activity and osteocalcin production

C2C12 or 2T3 cells were seeded at a density of 2 × 10^4^/cm^2^ in medium containing 10% FBS. The following day, before treatment, the medium was replaced with 5% serum-containing medium. Cells were lysed in 100 mM glycine, 1 mM MgCl_2_, and 1% Triton X-100 at pH 10. AP activity in the cell lysate was determined using a buffer containing 2-amino-2-methylpropanol and *p*-nitrophenylphosphate (Sigma-Aldrich. Inc.). The amount of osteocalcin secreted in the medium was determined using an ELISA kit (Biomedical Technologies, Inc., Stoughton, MA, USA). Both activities were normalized for total protein concentration, determined using a Bio-Rad DC protein assay kit.

### Mineralization assay

Freshly isolated murine bone marrow cells pooled from three mice were seeded on 12 well tissue culture plates at 5 × 10^6^ cells per well in standard culture medium and cultured for 10 days. Half the medium was replaced every 5 days. Calvaria cells isolated from adult mice were seeded at 0.02 × 10^6^ cells per well and cultured for 3 days. FBS then was reduced to 2%, and 50 ng/mL BMP-2 was added in the presence or absence of 10^−8^ M E_2_ in both types of cells. Two days later, 10 mM β-glycerolphosphate was added to the medium. The mineralization matrix was stained with 40 mM alizarin red solution 2 weeks later.

### Quantitative RT-PCR

Total RNA was extracted using Ultraspec (Biotecx Laboratories, Houston, TX, USA) and reverse-transcribed using the High-Capacity cDNA Archive Kit (Applied Biosystems) according to the manufacturer's instructions. Taqman quantitative reverse-transcriptase polymerase chain reaction (RT-PCR) was performed as described previously.(36) The primers and probes for murine Smad6 and rRNA18S were manufactured by the TaqMan Gene Expression Assays service (Applied Biosystems). Gene expression was quantified by subtracting the rRNA18S threshold cycle (*C*_*t*_) value from the *C*_*t*_ value of the gene of interest and expressed as 2−Δ*C*_*t*_, as described by the protocol of the manufacturer.

### Other assays

Intracellular ROS were quantified with dichlorodihydrofluorescein diacetate dye,(37) using bone marrow cells flushed from femurs and washed with PBS. Glutathione reductase activity was assayed with a kit from Cayman Chemical Company (Ann Arbor, MI, USA). Apoptosis in cultured cells was determined by measuring caspase-3 activity by cleavage of the fluorogenic substrate Ac-DEVD-AFC (Biomol, Plymouth Meeting, PA, USA), as described previously.(38)

### Statistical analysis

ANOVA was used to detect effects of various in vivo and in vitro treatments after establishing that the data were normally distributed and equivalency of variances. Bonferroni's method was used to perform appropriate pairwise comparisons of treatment groups. In cases where one or both of the requirements for performing ANOVA were not met, Kruskal-Wallis ANOVA on ranks was used, followed by Dunn's method, to perform pairwise comparisons of treatment groups. Unless otherwise stated, results are presented as mean ± SD and in vitro assays performed in triplicate and repeated at least one time.

## Results

### ERα NERKI is capable of mediating the antiapoptotic effects of E_2_ on osteoblasts

To investigate whether direct binding of the ERα to DNA is required for the ability of this receptor to mediate the effects of estrogens on the lifespan of bone cells in vivo, we first investigated whether the ERα NERKI was capable of mediating the known antiapoptotic effects of estrogens on osteoblasts in vitro and in vivo. In agreement with previous findings from our group,(6,25) we found that E_2_ prevented apoptosis of osteoblasts induced by H_2_O_2_ or the topoisomerase inhibitor etoposide as effectively in osteoblastic cells isolated from calvaria of *ERα*^*NERKI*/−^ mice, as it did in cells from WT mice (1*A*). In line with these in vitro effects, the prevalence of osteoblast apoptosis was increased in vivo in both WT and *ERα*^*NERKI*/−^ mice 6 weeks following OVX, as determined by in situ end labeling of vertebral sections (see 1*B*). Moreover, E_2_ replacement prevented the OVX-induced increase in osteoblast apoptosis in both WT and *ERα*^*NERKI*/−^ mice.

**Fig. 1:**
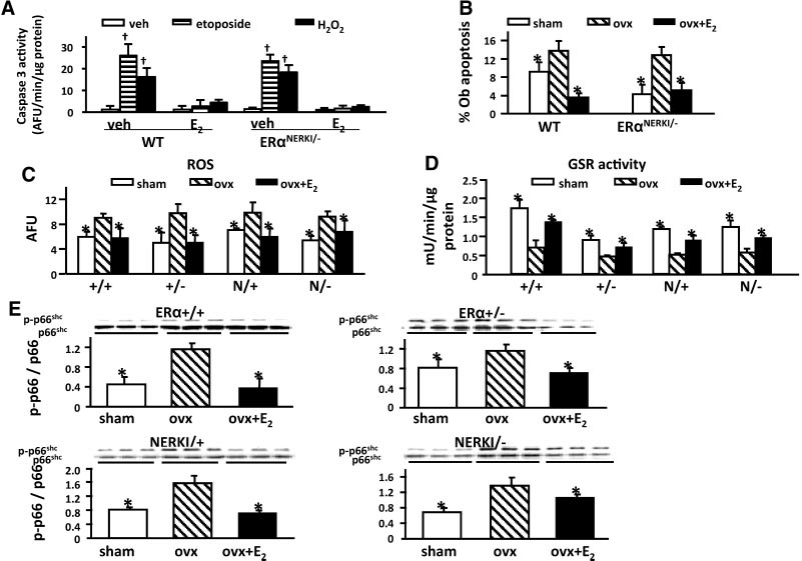
E_2_ effects on osteoblast apoptosis, ROS levels, GSR activity, and p66^shc^ phosphorylation are preserved in *ERα^NERKI^*^/−^ mice. (*A*) Caspase-3 activity in calvaria cells isolated from wild-type (WT) control or *ERα^NERKI^*^/−^ mice pretreated with vehicle (veh) or E_2_ (10^−8^ M) for 1 hour. Cells then were treated with veh, etoposide (5 × 10^−5^ M), or H_2_O_2_ (5 × 10^−5^ M) for 6 hours. (*B*) Osteoblast apoptosis determined by in situ end labeling in sections of undecalcified vertebral bone (L1–5) of 5-month-old WT or *ERα^NERKI^*^/−^ mice sham operated or OVX. OVX animals received veh or E_2_ replacement for 6 weeks (*n* = 4 to 6 animal/group). (*C*) ROS levels and (*D*) GSR activity in bone marrow cells from 5-month-old WT, *ERα*^+/−^, *ERα^NERKI^*^/+^, and *ERα^NERKI^*^/−^ mice sham operated or OVX and treated as described in *B* (*n* = 4 animals/group). (*E*) Phosphorylation of p66^shc^ determined by Western blot analyses in vertebral lysates from the same mice as in *C*; each lane represents one animal. ^†^*p* < .05 versus respective vehicle; *p < .05 versus OVX.

### Estrogens suppress oxidative stress in vivo independently of the DNA-binding function of the ERα

We next compared the effects of OVX and E_2_ replacement on ROS levels and GSR activity in the bone marrow and on p66^shc^ phosphorylation in vertebral lysates in WT and *ERα*^*NERKI*/−^ mice 6 weeks after the hormonal manipulation (see Fig. 1*C, D*). We also compared these changes in mice haploinsufficient for ERα (*ERα*^+/−^) and mice in which one copy of the ERα has been replaced by the *NERKI* mutant (*ERα*^*NERKI*/+^). As we had seen in our earlier work, ROS levels were increased (see 1*C*) and GSR activity was decreased (see 1*D*) following OVX, and these changes were prevented by E_2_ replacement in WT animals. Similar results were obtained in *ERα*^+/−^, *ERα*^*NERKI*/+^, and *ERα*^*NERKI*/−^ mice, indicating that the antioxidant properties of estrogens are independent of the DNA-binding function of the ERα.

We also observed an increase in p66^shc^ phosphorylation 6 weeks following OVX and its reversal by E_2_ replacement in WT, *ERα*^+/−^, *ERα*^*NERKI*/+^, and *ERα*^*NERKI*/−^ mice, demonstrating that the negative regulation of p66^shc^ phosphorylation by estrogens in vivo is mediated via a mechanism that does not require ERα binding to DNA (see 1*E*). The increase in p66^shc^ phosphorylation in vertebral lysates after OVX was reproduced in both WT and *ERα*^*NERKI*/−^ mice in a second experiment in which the mice were sacrificed 5 days after OVX (data not shown).

### ERα^NERKI/−^ mice exhibit a decrease in osteoblastogenesis

To ascertain the requirement or lack thereof of direct binding of the ERα to DNA for the suppressive effect of estrogens on osteoblastogenesis, we proceeded with a comparison of osteoblastogenesis in WT and *ERα*^*NERKI*/−^ mice by quantifying the number of progenitors able to form CFU-Fs and CFU-OBs in ex vivo bone marrow cultures seeded at three different densities (2*A*). The number of CFU-Fs was similar among all four genotypes. However, the number of CFU-OBs was significantly decreased in the bone marrow cultures from both *ERα*^*NERKI*/+^ and *ERα*^*NERKI*/−^ mice compared with the WT mice, independent of the initial seeding density. In addition to the decrease in numbers, CFU-OB colonies from *ERα*^*NERKI*/+^ and *ERα*^*NERKI*/−^ mice were smaller and displayed irregular shapes compared with cultures from WT or *ERα*^+/−^ mice, suggesting a defective osteoblast differentiation process analogous to the situation we had described previously in SAMP6 mice.(39) (see 2*B*). To determine whether the DNA-binding domain of the ERα is required for normal development of osteoblast progenitors from mesenchymal stem cells, we examined CFU-OBs derived from *ERα*^−/−^ mice. The shape and number of colonies from ERα^−/−^ mice were indistinguishable from those of WT mice, indicating that normal CFU-OBs are formed in the absence of the ERα (see 2*C*).

**Fig. 2:**
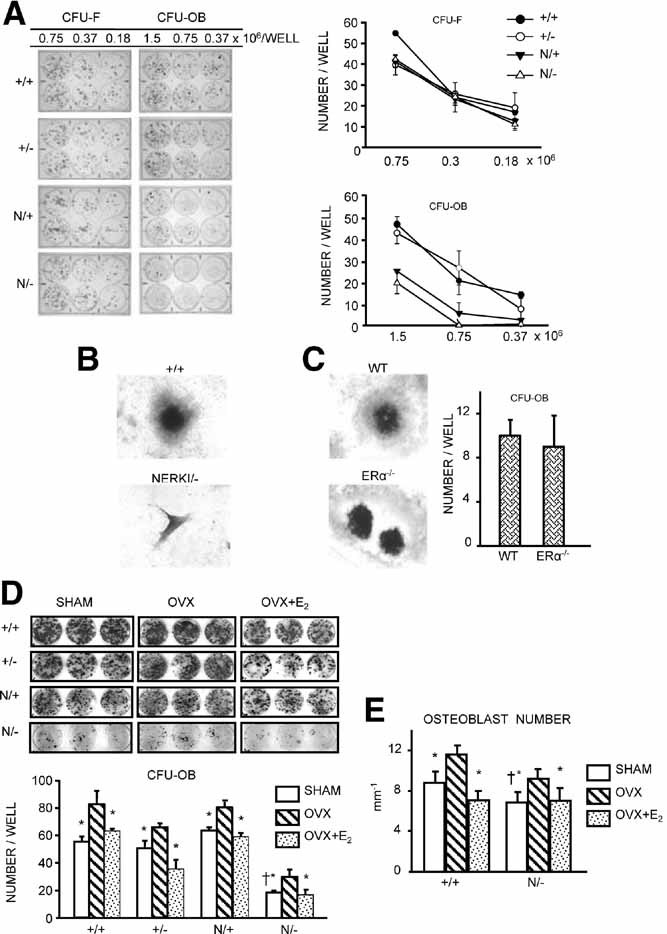
Osteoblastogenesis is decreased in *ERα^NERKI^*^/−^ mice. (*A*) CFU-Fs or CFU-OBs in the bone marrow from femora of intact mice of the indicated genotypes. Cells from three mice were pooled and plated in duplicate at three different densities for each genotype. CFU-Fs were stained for alkaline phosphatase after 10 days, and CFU-OBs were stained with von Kossa to detect mineral after 25 days (*left panel*). The graphs on the right represent the quantification of CFUs depicted on the left. (*B*) Photomicrographs show representative CFU-OB colonies (50×) obtained from WT (*ERα*^+/+^) or *ERα^NERKI^*^/−^ mice or (*C*) from WT or *ERα*^−/−^ mice; +/+ and WT refer to the respective littermate controls, as detailed in “Materials and Methods.” The graph on the right represents the quantification of CFU-OBs depicted on the right. (*D*) CFU-OBs obtained from femora of mice used in the experiment described in Fig. 1C. Cells from three mice were pooled and plated in triplicate at 10^6^ cells per well. The graph on the bottom represents the quantification of CFU-OBs depicted on the top. (*E*) Osteoblast numbers on longitudinal undecalcified sections of L1–4 vertebrae from mice used in the experiment described in Fig. 1C (*n* = 6 animals per group). *p < .05 versus OVX; ^†^*p* < .05 versus +/+ sham.

Having established a difference in CFU-OB numbers under basal conditions among the four genotypes, in a second experiment, we went on to investigate the effect of estrogen manipulation in the different genotypes (see 2*D*). As in the preceding experiment, *ERα*^*NERKI*/−^ mice exhibited strikingly fewer CFU-OBs than WT and *ERα*^+/−^ mice. In this experiment, CFU-OBs from *ERα*^*NERKI*/−^ mice were discernibly decreased compared with *ERα*^*NERKI*/+^ mice. Nonetheless, all four genotypes exhibited an increase in CFU-OB numbers following OVX, and the OVX-induced increase was prevented in all four genotypes by E_2_ replacement. In agreement with these results, the number of osteoblasts in vertebral cancellous bone also was decreased in sham operated *ERα*^*NERKI*/−^ mice compared with WT sham-operated controls. More important, the number of osteoblasts from *ERα*^*NERKI*/−^ mice was increased following OVX, and this was prevented by E_2_, just as was observed in WT mice (see Fig. 2E).

### ERα^NERKI/−^ mice have low basal BMD and lose cancellous bone following OVX

*ERα*^*NERKI*/−^ mice exhibited low bone mass at baseline in both femur and spine (3*A*). These mice also exhibited a decrease in vertebral length and volume, as well as femoral bone area (3*B*). Interestingly, when comparing mice carrying two copies of the WT ERα (*ERα*^+/+^) or one copy (*ERα*^+/−^) or the ERα *NERKI* mutant together with the WT ERα (*ERα*^*NERKI*/+^) or alone (*ERα*^*NERKI*/−^), there was a decrease at baseline BMD in the femur and spine of *ERα*^+/−^, *ERα*^*NERKI*/+^, and *ERα*^*NERKI*/−^ mice compared with *ERα*^+/+^ mice. In agreement with the dual-energy X-ray absorptiometry (DXA) results, unstained longitudinal sections of bone viewed in dark field confirmed the decreased vertebral size and diminished cancellous and cortical bone in the *ERα*^*NERKI*/−^ mice (see 3*C*). 3D BMD, cortical thickness, and trabecular thickness, as determined by µCT, also were decreased in the *ERα*^*NERKI*/−^ mice (but trabecular number and trabecular separation were indistinguishable) compared with WT controls under basal conditions (Table 1). Moreover, similar to the WT controls, the *ERα*^*NERKI*/−^ mice lost cancellous (see 3*D*) but not cortical (see 3*E*) bone following OVX. Nonetheless, E_2_ replacement at the dose used in our study did not prevent the loss of cancellous bone. A similar phenomenon was observed in the studies of Syed and colleagues,(40) but cancellous bone loss in the *ERα*^*NERKI*/−^ mice was prevented by a higher dose of E_2_ replacement in that earlier study.

**Fig. 3:**
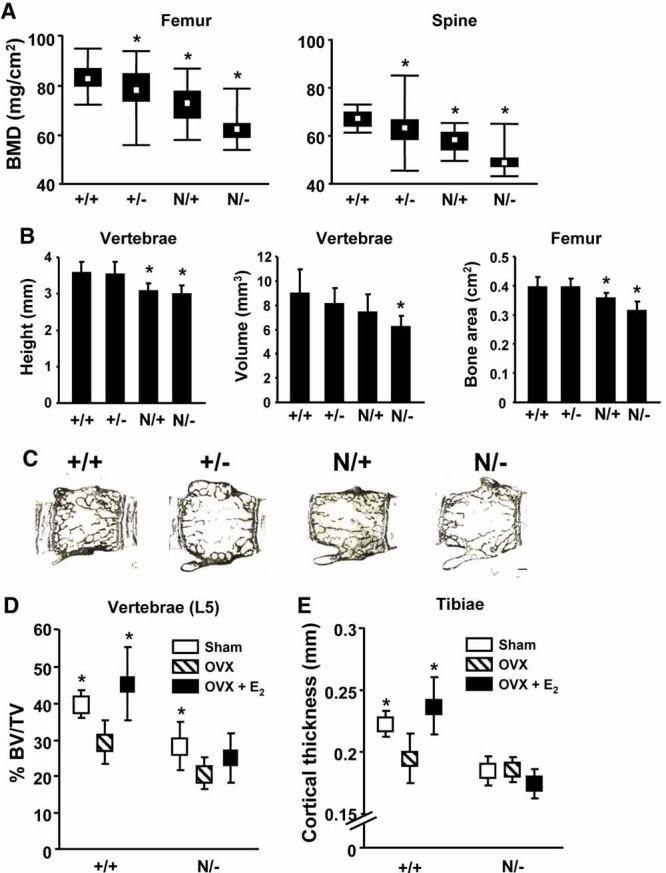
*ERα^NERKI^*^/−^ mice have low bone mass and lose cancellous bone following OVX. (*A*) Femoral and spinal BMD determined in 5-month-old mice of the indicated genotypes using Piximus (*n* = 26 to 31 per group). The box indicates the interquartile range around the median (*white square inside the box*), and the vertical lines represent values plus or minus 1.5 times the interquartile range. (*B*) Vertebral dimensions and femoral bone area were determined in mice described in *A* using a micrometer and Piximus, respectively (*n* = 7 to 11 per group). (*C*) Representative photomicrographs of lumbar vertebrae from three mice per genotype, unstained and viewed at 25× without coverslips. (*D*) Vertebral cancellous bone volume (BV/TV) and (*E*) cortical thickness of tibiae as determined by µCT in *ERα*^+/+^ or *ERα^NERKI^*^/−^ mice from the experiment described in 1*C* (*n* = 7 to 11 per group). *p < .05 versus +/+; ^†^*p* < .05 versus OVX.

**Table 1:** µCT Measurements of Lumbar Cancellous Bone (L5) and Mid-Diaphyseal Tibial Cortical Bone in *ERα*^+/+^, *ERα*^+/−^, *ERα*^*NERKI*/+^, and *ERα*^*NERKI*/−^ Female Mice

µCT Measurements	*ERα*^+/+^	*ERα*^+/−^	*ERα*^*NERKI*/+^	*ERα*^*NERKI*/−^
3D-BMD (mg HA/cm^3^)	298.9 ± 24.7	238.0 ± 48.4	271.1 ± 61.3	200.5 ± 48.7*
BV/TV %	39.5 ± 3.8	32.3 ± 6.2	37.7 ± 9.4	28.0 ± 6.6*
Trabecular number (per mm)	4.98 ± 0.32	4.56 ± 0.57	5.31 ± 0.96	4.79 ± 0.56
Trabecular thickness (µm)	70.7 ± 2.7	65.5 ± 5.7	64.5 ± 5.8	56.9 ± 5.5*
Trabecular separation (µm)	209.8 ± 16.8	224.3 ± 32.6	200.5 ± 34.4	209.6 ± 27.9
Cortical thickness (mm)	0.22 ± 0.011	0.21 ± 0.012	0.21 ± 0.016	0.19 ± 0.012*

* *p* < .05 versus *ERα*^+/+^ controls, *n* = 7–11.

### E_2_ attenuates BMP-2-induced osteoblast differentiation and the expression of BMP-2 target genes via ERα

Prompted by the evidence from the *ERα*^*NERKI*/−^ mouse suggesting that estrogens attenuate osteoblastogenesis independently of the ability of ERα to interact directly with DNA, we went on to investigate whether estrogens attenuate osteoblastogenesis via a cell autonomous mechanism and whether such an effect is exerted via an extranuclear action of the ERα mediated through the activation of cytoplasmic kinases. To establish the generality of such a mechanism, we used several cell models: two different established murine cell lines, a human osteosarcoma cell line with conditional expression of the ERα (U2OS-ERα), and primary cultures of osteoblast progenitors obtained from calvaria or the bone marrow of C57BL/mice or *ERα*^*NERKI*/−^ mice. As shown in 4*A*, BMP-2 dose dependently stimulated the differentiation of the preosteoblast cell line 2T3, as determined by AP activity. This effect was noticeable as early as day 1, reached a peak at day 3, and decreased by day 5. Addition of E_2_ attenuated the effect of BMP-2. Similarly, BMP-2 stimulated osteoblast differentiation in the uncommitted mesenchymal progenitor cell line C2C12 with a maximal effect at 3 days of culture, and E_2_ attenuated the effect of BMP-2 (see 4*B*). Consistent with its suppressive effect on BMP-2-induced osteoblast differentiation, E_2_ also attenuated the stimulating effect of BMP-2 on the secretion of osteocalcin, an osteoblast-specific biosynthetic product, at days 3 and 5 of the cultures (see 4*B*). Moreover, E_2_ antagonized BMP-2-induced gene transcription in C2C12 cells, as determined by the expression of the BMP-2 target gene *Smad6* (see 4*C*).

**Fig. 4:**
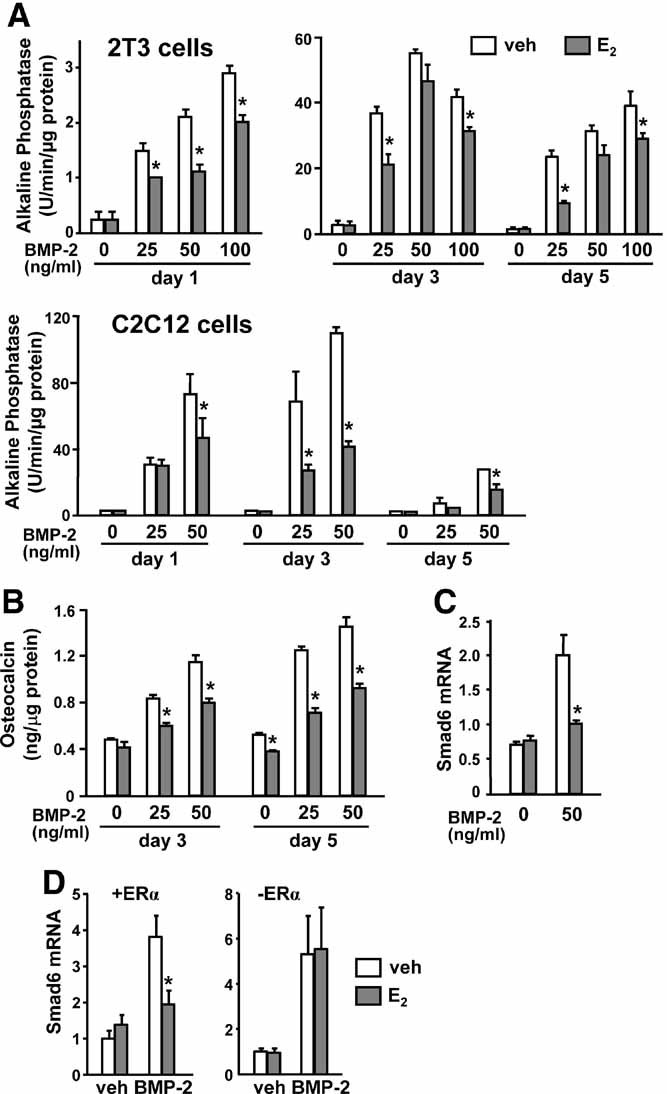
E_2_ attenuates BMP-2-induced osteoblast differentiation and target gene expression. (*A*) Alkaline phosphatase activity in 2T3 and C2C12 cells incubated with vehicle or the indicated doses of BMP-2 in the presence of E_2_ (10^−8^ M) for 1, 3, and 5 days. (*B*) Osteocalcin levels in the culture medium of C2C12 cells treated as described earlier for 3 and 5 days. (*C*) *Smad6* mRNA levels determined by quantitative RT-PCR in C2C12 cells treated as described earlier for 3 days or (*D*) in U2OS cells pretreated with vehicle or E_2_ (10^−7^ M) for 1 hour and treated with or without BMP-2 (100 ng/mL) for 2 hours in the presence (*left panel*) or absence (*right panel*) of doxycycline. *p < .05 versus BMP-2.

To establish that the effect of E_2_ on BMP-2-induced transcription was indeed mediated via the ERα, we examined the effects of E_2_ on BMP-2-induced *Smad6* mRNA in the human osteoblast-like osteosarcoma cell line U2OS stably expressing doxycycline-inducible ERα. BMP-2 stimulated *Smad6* transcription in U2OS cells both in the absence or presence of ERα. However, whereas E_2_ attenuated the effect of BMP-2 in U2OS cells expressing the ERα, the effect of E_2_ was abolished in cells lacking the ERα (see 4*D*).

**Fig. 5:**
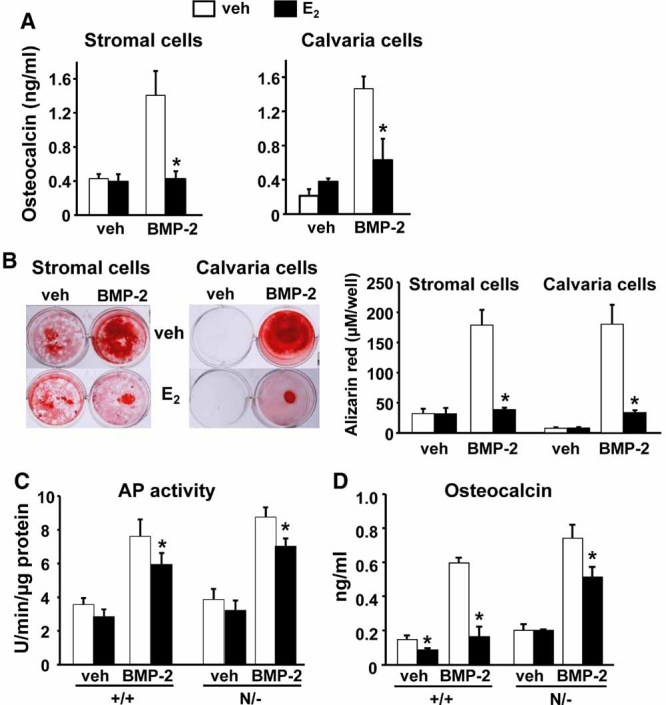
BMP-2-induced osteoblast differentiation and mineralization is decreased by E_2_ in primary osteoblastic cell cultures. (*A*) Osteocalcin levels in the medium and (*B*) mineralized matrix visualized following alizarin red staining (*left panel*) and quantified after extraction (*right panel*) of bone marrow– or calvaria-derived cells treated with BMP-2 (25 ng/mL), E_2_ (10^−8^ M), or E_2_ and BMP-2 in the presence of β-glycerophosphate for 18 days. (*C*) Alkaline phosphatase activity in parallel cultures of calvaria-derived osteoblasts from WT controls (*ERα*^+/+^) and *ERα^NERKI^*^/−^ mice incubated with BMP-2 (25 ng/mL), E_2_ (10^−8^ M), or E_2_ and BMP-2 for 3 days. (*D*) Osteocalcin levels in the culture medium of same cells as in *C* treated as described for 10 days. *p < .05 versus BMP-2 alone. The same results were reproduced in a second experiment.

The findings with the established cell lines were readily reproduced in primary cultures of bone marrow–derived stromal cells or primary cultures of calvaria-derived cells, models that more closely represent normal osteoblastic cells in vivo. As expected, addition of BMP-2 to these primary cultures strongly promoted osteoblast differentiation and maturation, as determined by osteocalcin secretion (5*A*) and mineralization (5*B*). In full agreement with the evidence from the cell lines, E_2_ decreased BMP-2-induced AP and osteocalcin secretion as well as mineralization in both the bone marrow– and the calvaria-derived primary osteoblastic cell cultures (see Fig. 5). Moreover, practically identical results were obtained in cultures of calvaria cells from WT (*ERα*^+/+^) and the *ERα*^*NERKI*/−^ mice (see Fig. 5*C, D*), establishing that the DNA-binding function of ERα is indeed dispensable for the attenuating effect of estrogens on osteoblast differentiation.

### E_2_ stimulates Smad1 phosphorylation at the linker region and attenuates BMP-induced transcription via ERKs

Based on the finding that estrogens inhibit osteoblastogenesis in both WT and *ERα*^*NERKI*/−^ mice and cells, we next tested the hypothesis that the attenuating effect of E_2_ on BMP-2-induced transcription and osteoblast commitment/differentiation was mediated by the activation of cytoplasmic kinases, such as ERKs. As shown in 6*A*, E_2_ attenuated BMP-2-induced phosphorylation of Smad1/5/8 in the C2C12 cell model, in agreement with earlier studies of ours.(13) In addition, E_2_ attenuated the BMP-2-induced transcriptional activation of the *Smad6*-luciferase construct (see 6*B*). Importantly, the specific MEK inhibitor PD98059 reversed the attenuating effect of E_2_ on both BMP-2-induced Smad1/5/8 phosphorylation and activation of transcription. However, PD98059 by itself had no effect on the BMP-2-induced Smad1/5/8 phosphorylation or transcription, indicating that these effects of BMP-2 do not require ERK activation. Total Smad1 levels were not affected by any one of these treatments (data not shown).

**Fig. 6:**
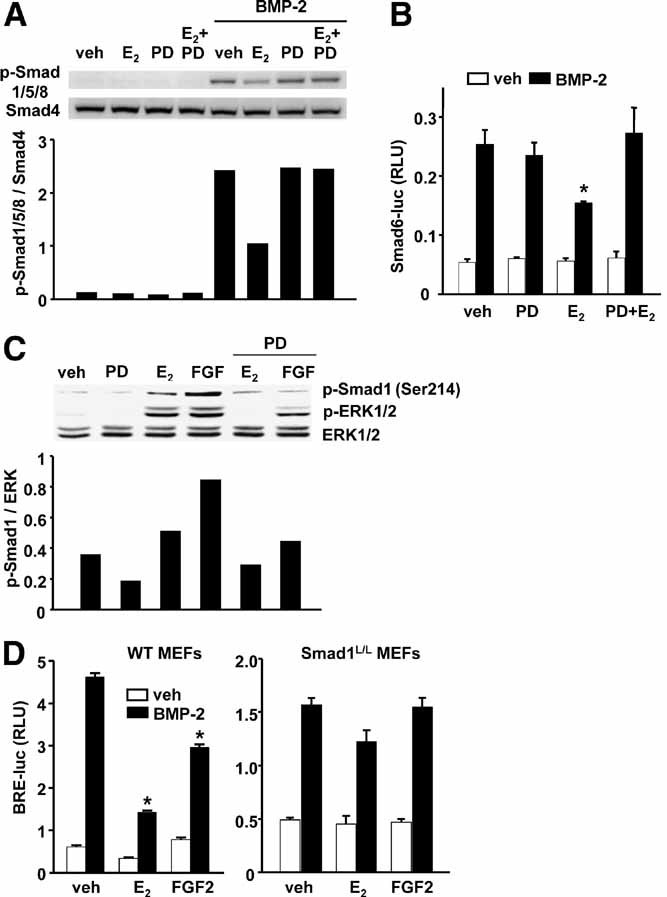
ERKs are required for the inhibitory actions of E_2_ on BMP-2-induced transcription. (*A*) Smad1/5/8 phosphorylation by Western blot analysis in C2C12 cells pretreated with vehicle, PD98049 (25 µM), or E_2_ (10^−8^ M) for 1 hour followed by vehicle or BMP-2 (50 ng/mL) for 1 hour. Bar graph represents the quantification of the intensity of the bands with an imaging system. (*B*) Luciferase activity in C2C12 cells transfected with a Smad6-Luc reporter construct and pretreated as in *A*, followed by treatment with vehicle or BMP-2 for 24 hours. (*C*) ERK1/2 and Smad1 (Ser214) phosphorylation by Western blot analysis in C2C12 cells pretreated with vehicle or PD98049 for 1 hour followed by E_2_ or FGF2 (5 ng/mL) for 15 minutes in serum-free medium. (*D*) Luciferase activity in MEFs transfected with a BMP response element reporter construct and pretreated for 1 hour with E_2_ or FGF2, followed by treatment with vehicle or BMP-2 (25 ng/mL) for 24 hours. *p < .05 versus BMP-2 alone.

Furthermore, E_2_ as well as FGF2, used here as a positive control, stimulated the phosphorylation of ERKs as well as the phosphorylation of Smad1 at p-Serine 214 in its linker region (see 6*C*). Ser214 phosphorylation is the direct result of ERK activation(23,41) and triggers Smad1 proteasomal degradation leading to a decrease in Smad1 transcriptional activity.(21,32) In agreement with this evidence, PD98059 prevented the ability of E_2_ to stimulate ERKs as well as Smad1 Ser214 phosphorylation. As expected, PD98059 also prevented FGF2-induced ERKs and Smad1 phosphorylation (see 6*C*). Finally, to verify that the MAPK sites in the linker region of Smad1 are important for the inhibitory actions of E_2_ on BMP-2 signaling, we used MEFs from WT or *Smad1*^*L*^*/*^*L*^ mice that carry a *Smad1* allele lacking all four MAPK sites in the linker region.(24) Addition of FGF2 to WT MEFs decreased BMP-2-dependent activation of a BMP transcriptional reporter construct, whereas addition of FGF2 to *Smad1*^*L/L*^ cells had no effect, as seen before by others.(21) Importantly, E_2_ also failed to inhibit BMP-induced transcription in *Smad1*^*L/L*^ cells (see 6*D*).

## Discussion

The evidence presented in this report illustrates that estrogens attenuate oxidative stress as well as the differentiation and apoptosis of osteoblasts by a nonclassical mechanism of ERα action. Specifically, our data reveal that the ability of estrogens to suppress oxidative stress and thereby attenuate the apoptosis of osteoblasts does not require binding of the ERα to DNA. The demonstration of estrogens' ability to suppress ROS levels and increase GSR activity in the bone marrow of *ERα*^*NERKI*/−^ mice strongly suggests that the dispensability of ERα binding to DNA for the antioxidant properties of estrogens extends beyond osteoclasts and osteoblasts and therefore must be a common mechanism of this property of estrogens in all their other target tissues.

**Fig. 7:**
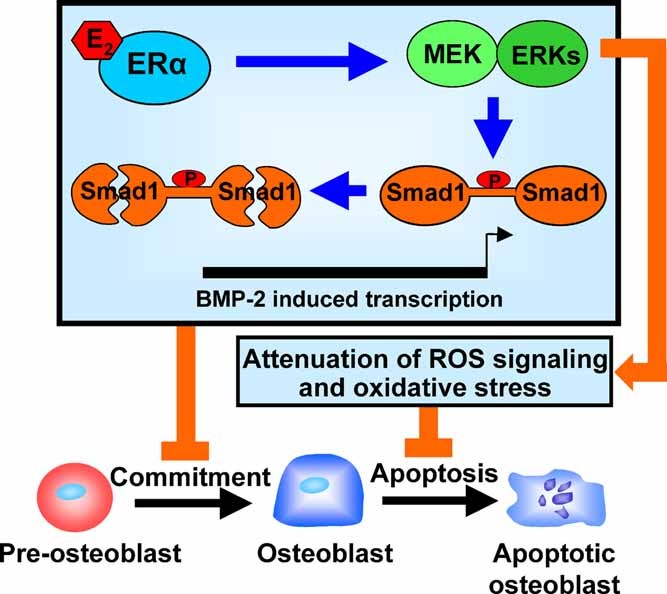
DNA-binding-independent actions of ERα on osteoblasts. Estrogens attenuate BMP-2-induced transcription by promoting the phosphorylation of Smad1 at its linker region, which, in turn, increases the proteasomal degradation of Smad1. This latter effect of the ERα results from the activation of ERKs. This mechanism contributes to the suppressive effect of estrogens on osteoblastogenesis and thereby osteoblast number and bone formation. A similar ERK-dependent decrease of ROS by estrogens is responsible for the antiapoptotic effect of these sex steroids on osteoblasts. The unleashing of this inhibitory effect, on loss of estrogens (e.g., menopause), is responsible for the increased osteoblast number and bone formation as well as the increase in osteoblast/osteocyte apoptosis that ensues following acute estrogen deficiency.

Using the *ERα*^*NERKI*/−^ mouse model, we have obtained compelling evidence that binding of ERα to DNA is also dispensable for the attenuating effects of estrogens on osteoblastogenesis. Moreover, using a variety of osteoblastic models from mice and humans, including primary cultures of calvaria- and bone marrow–derived osteoblastic cells, we demonstrate herein that E_2_ attenuates BMP-2-induced transcription and thereby osteoblastogenesis via ERK activation and downstream phosphorylation of Smad1 at the linker region of the protein, which leads to Smad1 proteasomal degradation. Consistent with this mode of action, E_2_ attenuated BMP-2-induced differentiation of primary cultures of calvaria- or bone marrow–derived osteoblastic cells from *ERα*^*NERKI*/−^ mice as effectively as in cells from WT littermates, establishing that the DNA-binding function of the ERα is indeed dispensable for this effect. These mechanisms are summarized in the model depicted in Fig. 7.

The evidence that estrogens attenuate osteoblast apoptosis and oxidative stress by an ERα-mediated mechanism that does not depend on the DNA-binding function of this receptor is consistent with extensive work of others showing that estrogens are indeed able to influence cells in their numerous reproductive and nonreproductive target organs, in part, by extranuclear actions of the ER involving kinase-mediated signaling.(42–44) In addition, the findings of this study are in agreement with earlier studies of ours showing that the suppressive effect of E_2_ on H_2_O_2_-induced phosphorylation of p66^shc^—a cumulative index of oxidative stress—is kinase-mediated and is inhibited by the ERK-specific inhibitor PD98059.(25)

ERβ is intact in *ERα*^*NERKI*/−^ mice, and therefore, we cannot categorically exclude the possibility that some of the effects of E_2_ on this model are mediated by ERβ. However, ERβ expression in murine bone is two to three orders of magnitude lower than ERα,(25) and osteoblast number and bone mass were unaffected in mice lacking ERβ.(45) In addition, studies in mice with a genuine null mutation of ERβ indicate that with the exception of impaired ovarian function, this isotype of the ER is not required in the mouse for the development and homeostasis of the major body systems.(46) In addition to regulating kinases, steroid receptors exert significant effects on gene expression without direct DNA binding, such as through transreppression of NF-κB or AP-1, leaving the possibility that the effects of estrogens on oxidative stress, osteoblastogenesis, and apoptosis may have resulted from protein-protein interaction of the ERα with either one of these transcription factors. Such an alternative scenario, however, is very unlikely in the case of NF-κB because this particular transcription factor inhibits BMP-2-induced osteoblastogenesis.(47)

Under basal conditions, *ERα*^*NERKI*/−^ as well as *ERα*^*NERKI*/+^ mice exhibited decreased numbers of CFU-OBs compared with WT controls, raising the possibility that the DNA-binding domain of ERα may be required for normal osteoblastogenesis. This clearly is not the case because CFU-OBs from mice lacking both *ERα* alleles were indistinguishable from WT controls. Consistent with the decreased osteoblastogenesis, cancellous osteoblast number was decreased in the *ERα*^*NERKI*/−^ mice compared to the *ERα*^+/+^ mice. In addition, DXA measurements showed a decrease in bone mineral content of the femur and spine of both *ERα*^*NERKI*/−^ and *ERα*^*NERKI*/+^ mice. The mechanistic basis of the effects of the *ERα*^*NERKI*/−^ mutant on basal osteoblastogenesis is unclear, and additional work, beyond the scope of this report, will be required to elucidate it. A possible explanation is that the ERα NERKI protein interferes with osteoblastogenesis via a function that is unique to this mutant protein, for example, binding and sequestering a protein normally required for the process. We also found that femoral and spinal BMD were decreased in the *ERα*^+/−^ as compared with *ERα*^+/+^ mice, in contrast to the observations of Smith and colleagues that osteopenia is not a consequence of the haploinsufficiency of the ERα in humans,(48) but we cannot account for this discrepancy.

The decreased BMD of the *ERα*^*NERKI*/−^ mice was confirmed by µCT measurements showing decreased BV/TV and trabecular thickness in the vertebrae, as well as decreased cortical thickness at the mid-diaphysis of the tibia compared with *ERα*^+/+^ mice. In agreement with our findings, Syed and colleagues(40) have reported previously that the *ERα*^*NERKI*/−^ mice have decreased cancellous BMD in multiple sites, but in their studies, cortical BMD was not different from that of the *ERα*^+/+^ mice. Also in agreement with this earlier work of Syed and colleagues, we found that *ERα*^*NERKI*/−^ mice lose bone mass (BV/TV) with OVX, but at the dose used in our study, estrogen replacement does not reverse this effect. However, in difference from the report of Syed and colleagues, we found that OVX had no discernible effect on cortical thickness, whereas they reported that cortical thickness increased with OVX. The reason for the difference is most likely because Syed and colleagues made their measurement 9 mm from the proximal end of the tibia. Using a fixed distance would place the measurement more distally in the *ERα*^*NERKI*/−^ mice, which have decreased tibia length compared with *ERα*^+/+^ mice. Furthermore, a more distal measurement would inexorably occur in the tibia-fibular junction and include the cortex of two bones, thus confounding interpretation of the measurement.(49)

A molecular explanation of the inhibitory effect of estrogens on osteoblastogenesis in vivo in both WT and *ERα*^*NERKI*/−^ mice has been provided in this report by the in vitro demonstration that E_2_ attenuates BMP-2-induced osteoblast differentiation and the transcription of BMP-2 target genes. Importantly, the attenuating effect of E_2_ on BMP-2-induced osteoblast differentiation in vitro was indistinguishable in cells from *ERα*^+/+^ and *ERα*^*NERKI*/−^ mice, strongly supporting the view that the DNA-binding function of the ERα is indeed dispensable for this effect. Specifically, we have obtained evidence that similar to earlier findings regarding the anti- and proapoptotic effects of estrogens on osteoblasts and osteoclasts, respectively, the attenuation of BMP-2-induced Smad1/5/8 phosphorylation is mediated via activation of ERKs and, more precisely, that E_2_ inhibits BMP-2 signaling by phosphorylating MAPK sites in the Smad1 linker region.

Similar to E_2_, other activators of MAPK, such as fibroblast growth factors (FGFs), have been shown to restrain BMP action during neural differentiation, limb development, and tooth formation.(50–52) Moreover, FGF or epidermal growth factor (EGF) inhibit BMP-induced gene expression and osteoblastogenesis in cell lines and primary human bone marrow–derived osteoblastic cells.(53–55) Phosphorylation of Smad1 by MAPK primes Smad1 for subsequent phosphorylation by GSK3β, which leads to Smad1 ubiquitination and degradation. Importantly, activation of Wnt signaling (i.e., inhibition of GSK3β) abrogates Smad1 degradation, thereby prolonging the duration of the BMP signal.(32) Thus Smad1 represents a site of convergence of both negative (e.g., MAPK and GSK3β) and positive (e.g., Wnt) regulatory signals of BMP-induced transcription. The convergence of multiple pathways on Smad1 underscores the importance of the modulation of the ERK-dependent phosphorylation of Smad1 by estrogens.

We had shown previously that activation of kinase-mediated actions of the ERα with synthetic ligands that selectively activate kinases without stimulating transcriptional activation results in increased osteoblast differentiation, whereas E_2_ did not. Interestingly, while both E_2_ and the synthetic ligands phosphorylated ERKs, only the latter inactivated GSK3β and stimulated TCF-mediated transcription.(8) The evidence of this study that E_2_ attenuates BMP-2-induced transcription is in agreement with our earlier observation that E_2_ is unable to activate canonical Wnt signaling—an inhibitor of Smad1 degradation. On the other hand, the ability of the synthetic ligands to stimulate osteoblastogenesis, whereas E_2_ could not, may be explained by the property of the former compounds to activate both ERK and Wnt signaling.

Different from the evidence reported herein, results of others from experiments with established cell lines (in some of which the level of ERα was artificially increased) or primary bone marrow stromal cell cultures have suggested that estrogens stimulate osteoblast differentiation, as evidenced by increased mineralization, alkaline phosphatase activity, and runx2 expression.(56–58) The discrepancy between the results of these earlier studies and ours may be due to the different experimental design. Indeed, we searched for the effects of estrogen on osteoblast differentiation in a setting in which primary bone marrow (or calvaria-derived cell) cultures and the BMP stimulus were used in combination. This combination was not used in those earlier in vitro studies. In support of this explanation, Usui and colleagues, in line with our findings, have observed an attenuating effect of estrogens on BMP-induced alkaline phosphatase activity in ROS17/2.8 osteoblastic cells.(11) More important, in agreement with the conclusions of this report, Usui and colleagues found that mice lacking Tob, an inhibitor of BMP, exhibit superenhancement of osteoblastic activity and augmentation of the bone-formation and mineral-apposition rates following loss of estrogens.

In summary, the composite evidence from the in vitro and in vivo experiments described herein demonstrates that estrogens exert cell autonomous effects on the differentiation and apoptosis of osteoblasts and provides a molecular explanation of the well-documented fact that following loss of estrogens in humans and mice, bone formation increases, albeit not in balance with the increased bone resorption, as does the prevalence of osteoblast and osteocyte apoptosis. In addition, the results of this report provide strong support of the view that the effects of estrogens on the birth and death of osteoblasts and osteocytes do not require the DNA-binding function of the ERα and result, in part, from antioxidant properties of these hormones.
